# Nitrogen optimization coupled with alternate wetting and drying practice enhances rhizospheric nitrifier and denitrifier abundance and rice productivity

**DOI:** 10.3389/fpls.2022.927229

**Published:** 2022-10-11

**Authors:** Abbas Ali Abid, Qichun Zhang, Muhammad Faheem Adil, Itrat Batool, Muhammad Abbas, Zeshan Hassan, Azhar Abbas Khan, Antonio Castellano-Hinojosa, Syed Hassan Raza Zaidi, Hongjie Di, Nader R. Abdeslsalam

**Affiliations:** ^1^Zhejiang Provincial Key Laboratory of Agricultural Resources and Environment, Key Laboratory of Environment Remediation and Ecological Health, Ministry of Education, Zhejiang University, Hangzhou, China; ^2^Department of Agronomy, College of Agriculture and Biotechnology, Institute of Crop Science, Zhejiang University, Hangzhou, China; ^3^Institute of Food Science, Chinese Academy of Agricultural Sciences, Beijing, China; ^4^National Engineering Laboratory for Improving Quality of Arable Land, Institute of Agricultural Resource and Planning, Chinese Academy of Agricultural Sciences, Beijing, China; ^5^College of Agriculture, Bahauddin Zakariya University, Layyah, Pakistan; ^6^Department of Soil and Water Sciences, Southwest Florida Research and Education Center, Institute of Food and Agricultural Sciences, University of Florida, Immokalee, FL, United States; ^7^Department of Agriculture Botany, Faculty of Agriculture, Saba Basha, Alexandria University, Alexandria, Egypt

**Keywords:** rice, water management, photosynthesis, slow-release fertilizer, nutrient management, nitrifier, denitrifier

## Abstract

Optimizing nitrogen (N) fertilization without sacrificing grain yield is a major concern of rice production system because most of the applied N has been depleted from the soil and creating environmental consequences. Hence, limited information is available about nutrient management (NM) performance at a specific site under alternate wetting and drying (AWD) irrigation compared to conventional permanent flooding (PF). We aimed to inquire about the performance of NM practices compared to the farmer’s fertilizer practice (FFP) under PF and AWD on rhizospheric nitrifier and denitrifier abundance, rice yield, plant growth, and photosynthetic parameters. Two improved NM practices; nutrient management by pig manure (NMPM); 40% chemical N replaced by pig manure (organic N), and nutrient management by organic slow-release fertilizer (NMSR); 40% chemical N replaced by organic slow-release N were compared. The results showed an increased total grain yield (16.06%) during AWD compared to PF. Compared to conventional FFP, NMPM, and NMSR significantly increased the yields by 53.84 and 29.67%, respectively, during AWD. Meanwhile, PF prompted a yield increase of 45.07 and 28.75% for NMPM and NMSR, respectively, (*p* < 0.05) compared to FFP. Besides, a significant correlation was observed between grain yield and nitrogen content during AWD (*R*^2^ = 0.58, *p* < 0.01), but no significant correlation was observed during PF. The NMPM contributed to photosynthetic attributes and the relative chlorophyll content under both watering events. Moreover, relatively higher abundances of ammonia-oxidizing bacteria (AOB) and ammonia-oxidizing archaea (AOA) were observed during AWD, and the highest value was found after the late panicle stage. Our results suggest that the AWD–NMPM model is the best option to stimulate nitrifier and denitrifier gene abundance and promote rice production.

## Introduction

The nutrient management (NM), at a specific site, is an approach for dynamic nutrient optimization in rice to overcome the supply and demand of nutrients (Rodriguez, 2020). The underlying premise of NM is to optimize nutrients applied to crops according to appropriate time and rate. The NM allows to calculate the required nitrogen (N), phosphorus (P), and potassium (K) amounts to attain a targeted yield, with additional rules for the timing of fertilizer applications, thereby providing concrete advice to farmers on the rate, sources, and time of fertilizer applications (Buresh et al., 2010; Yan et al., 2022). Nutrient management can improve crop yield and income. The NM approach in rice is well documented in terms of economic profitability and environmental impacts, but its effects on yield components are rarely studied.

Rice (*Oryza sativa* L.) is a staple food in many regions, including China, and a dynamic component of the world’s economy. According to the Agriculture Ministry of China (1990–2011), the average annual rice production and planting area have exceeded to 18.6 billion tons and 30.1 million hectares, respectively, and in 2020, China’s annual rice production was recorded as 211.86 million metric tons (Food and Agriculture Organization of the United Nations [FAO], 2020). About 95% of rice is grown under traditional submerged permanent flooding (PF) conditions, and about 50% of all the diverted freshwater is utilized by rice fields in China (Cai and Chen, 2010).

Meanwhile, the increased consumption of freshwater for domestic and industrial purposes has dwindled the water supplies available for irrigation purposes (Li and Li, 2010; Sun et al., 2012). This situation necessitates the utilization of water-saving irrigation strategies, including semi-dry cultivation, non-flooded mulching cultivation, intermittent irrigation, controlled irrigation, and alternate wetting and drying irrigation (AWD) (Shao et al., 2015; Cao et al., 2020). Among these, AWD is a very common and effective strategy (Yao et al., 2012). In AWD irrigation, the rice field is left to dry for a particular duration before being re-irrigated when the plants begin to display visual signs of water deficiency (Yao et al., 2012). Furthermore, N losses through surface runoff and leaching from flooded rice fields are significant (Liang et al., 2013), accounting for 60% of N loss during the growing period to the environment in the forms of ammonia, nitrate, and nitrous oxide, creating determinantal impacts on environment and human health (Gu and Yang, 2022). In contradiction to the permanent flooding (PF), AWD can save nutrients by 50–60% and water inputs by 20%, thereby increasing water use efficiency (WUE) by 36–55% (Zhang et al., 2009).

The N fertilizer plays a crucial role in optimal rice crop development; nevertheless, excessive N fertilizer usage in rice elevates production costs and has severe environmental consequences, such as soil degradation, eutrophication, water pollution, and emissions of greenhouse gases (Peng et al., 2010; Spiertz, 2010). The strategy of partially substituting chemical fertilizer with organic manure (or organic fertilization) can not only supply exogenous carbon (C), N, and other nutrients to avoid an insufficient supply of mineral N, but can also modulate the C/N ratio and N transformation processes (Huang et al., 2020). Therefore, more efficient N management practices must be adopted in rice to avoid excessive N fertilizer application (Xia et al., 2016). To deal with these challenges, it is critical to improve nitrogen use efficiency (NUE) (Guo et al., 2010). To enhance NUE, several N-saving methods, including balanced N fertilization, site-specific N management, and controlled/slow-release fertilizers, have been used (Jat et al., 2012; Sun et al., 2012), but their interactive effect on rice yield and nitrifier and denitrifier production is clueless.

Microbial-mediated nitrification and denitrification processes are critical for soil N equilibrium (Harter et al., 2014; Zhang and Ji, 2018). The various fertilization techniques, including manure application, have the capacity to affect nitrifier and denitrifier communities (Hallin et al., 2009; Yin et al., 2015). Ammonium (NH_4_^+^) is oxidized to nitrate (NO_3_^–^) via nitrite (NO_2_^–^) during nitrification, a process catalyzed by the ammonia monooxygenase (*amoA*) enzyme of ammonia-oxidizing bacteria (AOB) and archaea (AOA). Ammonium availability is thought to be the most important factor for the growth of AOB and AOA (Abid et al., 2018). The functional genes have been employed as biomarkers to examine N transformations in paddy soils (Francis et al., 2013; Levy-Booth et al., 2014), which have been further validated by evaluating functional N-genes including AOA, AOB, and nitrite reductase (*nirS*) genes (Chen et al., 2010; Yin et al., 2015). So, we aimed to count the nitrifying and denitrifying functional gene abundance engaged in N transformation.

It is well documented that about 20% of applied N is accumulated in the food chain, and the remaining diminishes due to high use of N fertilizers (Albiac, 2009). Interestingly, two-thirds of the N fertilizer could be saved in the rice system without compromising the rice grain yield (Wang et al., 2011). The high amount of N and water inputs do not guarantee increased N and water use efficiencies; therefore, the application frequencies must be optimized according to the crop requirements (Wang et al., 2007). A plethora of studies have focused on irrigated rice performance under AWD (Zhang et al., 2009) and different fertilization (Kiran et al., 2010; Zhang et al., 2011), but very limited information is available on synergetic effects of AWD and NM. Besides this, whether or not different types of fertilizer application rates and time in combination with different irrigation regimes prove advantageous to the grain yield needs further appraisal (Sun et al., 2012). Moreover, it is important to evaluate the management practices to be adjusted, rendering the water-saving strategies (Dong, 2008). In this context, the following objectives were devised to evaluate the performance of two different nutrient management practices: nutrient management by pig manure (NMPM) and nitrogen management by slow-release fertilizer (NMSR) compared to farmer fertilizer practice under AWD and PF irrigation on rice agronomic performance, grain yield, photosynthesis rate, and nitrifier and denitrifier gene abundance.

## Materials and methods

### Site description

Soil samples were collected from the upper surface (0–20 cm in depth) of a paddy field located in Hengxi Town, Ningbo City, Zhejiang Province, China (29°40′5″N; 121°35′41″E). Ningbo is situated on China’s southeast coast, in the southern part of the Yangtze River Delta. The study area has a subtropical monsoon climate, with an average annual temperature of 16.4°C, average annual precipitation of 1,480 mm, and average sunshine duration of 1,850 h. According to China’s soil classification system, the soil is categorized as red soil. The collected soil was air-dried, sieved to 2 mm, and stored for analysis. The soil was characterized as loam soil (33% sand, 42% silt, and 25% clay). The soil properties were as follows: pH (H_2_O) 4.53, available P 323.09 mg kg^–1^, available K 213.67 mg kg^–1^, organic matter 34.55 g kg^–1^, NO_3_^–^–N 7.94 mg kg^–1^, NH_4_^+^–N 5.29 mg kg^–1^, and cation exchange capacity (CEC) 14.58 cmol kg^–1^.

### Experimental design

A pot experiment was carried out on March 2020 in greenhouse of Zhejiang University, Zijingang Campus in Hangzhou, China, using a randomized complete block design (RCBD) with two watering events and three fertilizer treatments, and a control (no fertilizer addition). The hybrid rice variety Liangyou 0293, known as super hybrid variety due to high yield, was sown in pots (18 cm × 20 cm) to study gained biomass, crop yield, and photosynthesis. Twenty-four pots (four treatments × three replicates × two water events) with 30 cm diameter and depth of 35 cm were filled with two kg of soil. Different doses of fertilizers (NPK) were applied and saturated with deionized water and irrigation water collected from the study area. Rice seeds were sown in pots, and the pots were placed at ambient temperature (15–38°C) in a greenhouse. During the trial period, the soil was kept flooded by adding deionized water (around 3 cm above the soil surface).

The experiment was designed with four treatments including (1) CK; no fertilization, (2) farmer’s fertilizer practices (FFP); pig manure [moisture 21.32%, pH (1:2) 7.29, total organic C 26.23 g/kg, total organic matter 52.36 g/kg, total Kjeldahl N 4.12 g] 50 kg ha^–1^ (two-fraction mixture of urine, feces, and water) (6.8 g pot^–1^), urea 30 kg ha^–1^ (4.1 g pot^–1^) (50% of as basal, 50% at early panicle initiation stage), 50 kg ha^–1^ phosphorus was applied as single super phosphate (6.8 g pot^–1^) before transplanting. (3) 40% of chemical N replaced by organic N as nitrogen management by pig manure (NMPM); pig manure 146 kg ha^–1^ (19.9 g pot^–1^) before transplanting, urea 17.3 kg ha^–1^. 50% of urea as basal before transplantation, 50% at early panicle initiation stage (25% at early tillering and 25% at panicle initiation stages), single super phosphate (16% P_2_O_5_) 7.25 kg ha^–1^ before transplantation, and 8.4 kg ha^–1^ KCl were applied. Half of the potassium was applied before transplanting and the remaining half at the panicle initiation. (4) 40% of chemical N replaced by organic slow-release (SR) fertilizer (Sinotech, Beijing Development Co., Ltd.) N as nitrogen management by slow-release fertilizer (NMSR); SRF 13 kg ha^–1^ before transplantation (as basal), urea; 17.3 kg ha^–1^ (50% of as basal, 50% at early panicle initiation stage), KCl 8.4 kg ha^–1^ applied at panicle initiation stage and no addition of single super phosphate.

Two water levels were also used, that is, PF (permanent flooding by keeping water 3 cm throughout the trial period) and AWD (alternate wetting and drying irrigation). For AWD, water was kept at 3 cm for 10 days after transplanting (DAT, seeding) and then maintained at 0–3 cm during 11–24 DAT (tillering); subsequently, the soil remained dry until the white patches of the soil became visible (25–37 days, during later tilling stage), the reproductive period (from the end of panicle differentiation to flowering). Dry-wet irrigation management cycles were continued after 37 days (3 days 3 cm water and 3 days dry) until rice harvest.

### Determination of growth parameters

To determine the growth parameters, plants were harvested (roots were gently removed from the pots and washed manually with a gentle stream of water over a wire mesh sieve to remove soil media until roots were clean); then, root lengths (distance from the base of the stem to the tip of root) and shoot heights (distance from the crown of the stem to the tip of the uppermost leaf) were measured with a standard metric ruler (Mapodzeke et al., 2021). Plant root and shoot samples were dried at 70°C for 2 days in an air circulation oven (Jinghong, XMTD-8222) to ensure that a constant weight was reached for the determination of root/shoot dry biomass (Adil et al., 2020).

### Dry matter accumulation, N concentration, and yield components

The rice crop was harvested manually and threshed using a hand-driven thresher when it reached maturity (4 months following transplanting). After 3–5 days of air drying and subsequent grinding, the grain yield was determined. Plant samples were processed with deionized water before being sorted into roots, shoots, and panicles and oven-dried at 70°C. The ground plant samples (sieved by 2 mm mesh) were used for nutrient (N and C) content measurement using a Vario MAX CNS elemental analyzer after the samples were finely ground to pass through a 0.15 mm filter (Elementar Analysensystem GmbH, Hanau, Germany) (Golden et al., 2009).

### Measurement of relative chlorophyll content and photosynthetic attributes

Intact functional leaves (the third or fourth upper-most leaves) were selected to analyze photosynthetic parameters, that is, net photosynthetic rate (*Pn*), stomatal conductance (*Cond*), transpiration rate (*Tr*), and intercellular CO_2_ concentration (*Ci*), at maturity stage using an IRGA-based portable photosynthesis system (LI-6400; Li-COR, Lincoln, NE, United States). A built-in light-emitting diode source with a flow rate of 400 μmol s^–1^, relative humidity between 50 and 70%, and a CO_2_ concentration of 400 μmol mol^–1^ was used to calculate the photosynthetic parameters at a photon flux density of 1,000 μmol m^–2^ s^–1^. Besides, relative chlorophyll content as SPAD (Soil Plant Analysis Development) values was recorded using a chlorophyll (model ccm-200 plus). Furthermore, the chlorophyll content of leaves was determined using the acetone/ethanol combination technique (Chen and Chen, 1984).

### Determination of nitrifier and denitrifier gene abundance

The DNA of the soils was extracted from 0.5 g of fresh soil using Fast DNA SPIN Kit. The DNA extraction was done according to the manufacturer’s instructions (for soil; Bio 101, Carlsbad, CA, United States). Real-time quantitative polymerase chain reaction (qPCR) was used to determine the abundance of nitrifier and denitrifier genes (Abid et al., 2018, 2019). The qPCR was used to quantify nitrifiers and denitrifiers gene abundances. After DNA extraction, the concentration was measured by NanoDrop 2000 UV-Vis Spectrophotometer (Thermo Scientific), and its quality was assessed by 1% agarose gel electrophoresis. Primer pairs *amo*A1F (5′-GGG GTT TCT ACT GGT GGT-3′) and *amo*A2R (5′-CCC CTC KGS AAA GCC TTC TTC-3′), as well as *Crenamo*A23F (5′-ATG GTC TGG CTW AGA CG-3′) and *Crenamo*A616R (5′-GCC ATC CAT CTG TAT GTC CA-3′), were used to quantify bacterial and archaeal *amoA* genes, respectively. The abundance of *nirS* was detected using primers *nirS*-cd3aF (5′-GTS AAC GTS AAG GAR ACS GG-3′) and *nirS*-R3cd (5′-GAS TTC GGR TGS GTC TTG A-3′) (Throbäck et al., 2004), producing a 410-bp product in amplification conditions: 95°C for 3 min and 35 cycles of 15 s at 95°C, 30 s at 45°C, and 45 s at 72°C, with a final 5-min extension at 72°C (Supplementary Table 1). Reaction mixtures contained 10 μL GoTaq^®^ SYBR Green Master, Roche Diagnostics GmbH Mannheim, Germany, 200 μM of each primer, 2 μL of tenfold diluted DNA template (10–20 ng), and ultraclean water to 20 μL total volume. The plasmid of the N-related functional genes was extracted from DNA and serially diluted to generate a standard curve. The corresponding qPCR efficiency was 95–105%, and *r*^2^ was 0.99–1.00. The qPCR cycling conditions were the same for all targeted functional genes with 40 cycles.

### Statistical data analysis

To examine the significance of treatments, a two-way analysis of variance (ANOVA) test was performed to identify significant differences among the treatments. If the significant differences were identified at *p* < 0.05, a Tukey HSD *post hoc* test was applied followed by Duncan’s multiple range test (DMRT) to separate treatment means (Tang and Feng, 1997). The data were statistically analyzed using DPS9.50 (Data Processing System). The results were considered significant at *p* < 0.05. Origin Pro 2021 was used to create the graphs (Origin lab corporation, Wellesley Hills, Wellesley, MA, United States).

## Results

### Nitrogen and carbon concentration in root and shoot

The highest N content in root was recorded in FFP treatment regardless of the water events but comparatively higher during PF watering event (1.88%). Moreover, a comparatively lower N percentage in root was recorded in CK and NMSR (Table 1). Likewise, C concentration amounted highest in FFP treatment during AWD and in CK during PF. A dramatic increase in N was measured in MNSR treatment during PF compared to the same treatment NMSR during AWD which was 58.8% higher and showed water event had a significant effect on N content under NMSR. Contradictory to that, during PF, relatively higher N content was observed in CK as compared to NMSR. Interestingly, during PF, NMSR treatment showed a decline in N and C. In addition, C/N ratio was recorded as significantly higher in CK during both watering events that was two times greater than FFP treatment. Moreover, the interaction of root N was significant with fertilizer treatment (*p* < 0.00) but insignificant with water event and interactive effect of fertilizer treatment and water event, while C was significantly affected by water events and fertilizer treatments (Table 1). Differential response of watering events was observed in same fertilizer treatments.

**TABLE 1:** Combined effects of different fertilizer treatments during alternate wetting and drying (AWD) and permanent flooding (PF) on inorganic nutrients in root.

Water condition (W)	Treatment (T)	N (%)	C (%)	C/N ratio
AWD	CK	0.97 ± 0.02bc	33.66 ± 0.03ab	34.57 ± 0.46a
	FFP	1.74 ± 0.09a	35.98 ± 0.71a	20.74 ± 0.86cd
	NMPM	1.24 ± 0.03b	30.38 ± 0.51b	24.54 ± 1.01bc
	NMSR	1.16 ± 0.03a	35.13 ± 1.21ab	30.28 ± 1.46ab
PF	CK	0.96 ± 0.03a	35.03 ± 0.23ab	36.71 ± 1.09a
	FFP	1.88 ± 0.07c	31.44 ± 1.32ab	16.86 ± 1.39d
	NMPM	1.68 ± 0.11bc	33.67 ± 1.24ab	20.05 ± 0.74cd
	NMSR	0.81 ± 0.06c	24.08 ± 0.06c	29.86 ± 3.28ab
W		ns	**	**
F-value		1.48	82.91	13.61
*P*-value		0.24	0.000	0.0001
T		**	**	**
F-value		14.77	8.86	20.67
*P*-value		0.0014	0.0011	0.000
W*T		ns	*	ns
F-value		1.15	23.95	2.08
*P*-value		0.36	0.01	0.14

The identical letters inside a column show that there is no significant difference 95% probability. ns, the relationship between treatment and water condition is insignificant.

*Significant interaction between treatment and water condition at *P* ≤ 0.05 level.

**Highly significant interaction between treatment and water condition at *P* ≤ 0.01 level.

During AWD, the highest N concentration (2.21% N) in shoot was measured in FFP treatment which was more than double compared to CK treatments (Table 2), while the lowest concentration in shoot was measured in CK (0.80% N). Besides, the highest concentration was measured in NMSR during PF, which showed that NMSR treatment promoted N concentration in PF, whereas the same treatment reduced N concentration during AWD compared to other FFP and NM treatments. Of note, N concentration in fertilizer treatments (FFP, NMPM, and NMSR) during PF event was higher than AWD, showing that PF promoted N concentration compared to AWD. Set against CK, N was significantly increased by 2–3 times in AWD. In PF, N was dramatically increased in those treatments with N addition compared with CK. The C concentration in shoot was significantly higher in NMPM and NMSR treatments (Table 2). Approximately similar values of C were calculated in both water events and no difference in trends was observed in fertilizer treatments, except for a slightly higher value in NMPM and NMSR treatments. Compared to CK, fertilizer treatments promoted C concentration. The C/N ratio was observed higher in CK treatment during PF which was 2–3 times higher than in fertilizer treatments. The interaction of shoot N was significant with water event (*p* < 0.00), while shoot C interaction was significant with both water event (*p* < 0.001) and fertilizer treatment (*p* < 0.05). Both shoot N and C had insignificant interaction with interactive effect of water event and fertilizer treatment.

**TABLE 2:** Combined effects of different fertilizer treatments during alternate wetting and drying (AWD) and permanent flooding (PF) on inorganic nutrients in shoot.

Water condition (W)	Treatment (T)	N (%)	C (%)	C/N ratio
AWD	CK	0.80 ± 0.06c	37.94 ± 1.08a	47.77 ± 2.92a
	FFP	2.21 ± 0.06ab	40.01 ± 0.75a	18.04 ± 0.24bc
	NMPM	1.79 ± 0.08b	41.09 ± 0.86a	23.01 ± 1.21b
	NMSR	1.73 ± 0.13b	40.45 ± 0.47a	51.05 ± 3.78a
PF	CK	0.78 ± 0.07c	38.76 ± 0.84a	50.16 ± 4.84a
	FFP	2.71 ± 0.09a	40.072 ± 0.59a	14.82 ± 0.75c
	NMPM	2.48 ± 0.15a	41.07 ± 0.53a	16.61 ± 0.97bc
	NMSR	2.74 ± 0.15a	40.33 ± 0.38a	14.80 ± 0.81c
W		**	**	**
F-value		52.21	100.74	8.16
*P*-value		0.000	0.000	0.0016
T		ns	*	ns
F-value		0.75	29.05	0.18
*P*-value		0.45	0.01	0.91
W*T		ns	ns	**
F-value		1.58	2.98	24.02
*P*-value		0.297	0.201	0.000

The identical letters inside a column show that there is no significant difference 95% probability. ns, the relationship between treatment and water condition is insignificant.

*Significant interaction between treatment and water condition at *P* ≤ 0.05 level.

**Highly significant interaction between treatment and water condition at *P* ≤ 0.01 level.

### Plant growth contributing characters

**FIGURE 1:**
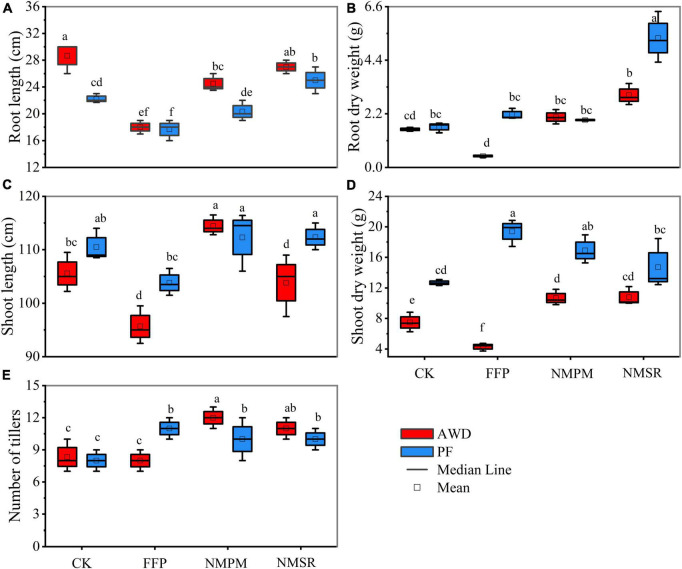
Combined effects of different fertilizer treatments during alternate wetting and drying (AWD) and permanent flooding (PF) on dry matter accumulation, that is, **(A)** root length, **(B)** root dry weight, **(C)** shoot length, **(D)** shoot dry weight, and **(E)** number of tiller. The arrow bar above the line shows standard error. The different letters show significant difference, while the same letters represent no significant differences among the treatments.

Plants showed a varying response to the treatments and water events. During AWD, significantly higher root length was observed in CK treatment followed by NMSR treatment, and the values were 29.21 and 27.3 cm, respectively. Meanwhile, during PF, the highest root length was observed in NMSR treatment followed by CK (Figure 1A). The least root length was measured in FFP which showed a negative effect on root length. During AWD, it showed a sequence of CK > NMSR > NMPM > FFP, whereas, during PF, the sequence was NMSR > CK > NMPM > FFP (Figure 1A). Generally, the root length was significantly higher in AWD than in PF, irrespective of the fertilizer treatments, while shoot length was found significantly higher in NMPM in both water events followed by NMSR and CK (Figure 1C). The highest value of shoot lengths in NMPM was 114.14 and 112.3 cm during AWD and PF, respectively. The root dry weights were observed higher in NMSR during both water events but relatively higher in PF than AWD (Figure 1B). The same trends were observed for shoot weight during AWD but during PF the highest value was recorded in FFP (Figure 1D). The number of panicles was significantly higher in NMPM and NMSR than in CK and FFP, mentioning that a reduced amount of pig manure and N was more efficient than the high application rate (Figure 1E). While comparing different water events, the number of panicles was observed significantly higher in CK, NMPM, and NMSR under AWD than PF. In general, relatively higher root length and number of panicles were observed during AWD events than PF, except for FFP which showed opposite response, while the dry shoot weight was relatively higher in PF. Dramatic root and shoot growth inhibition was instigated by FFP treatment during PF, where shoot growth was much more affected than root growth. Interestingly, NMSR treatment did not result in any further inhibition of root and shoot growth compared with other treatments.

### Correlation of N with plant dry matter accumulation

**FIGURE 2:**
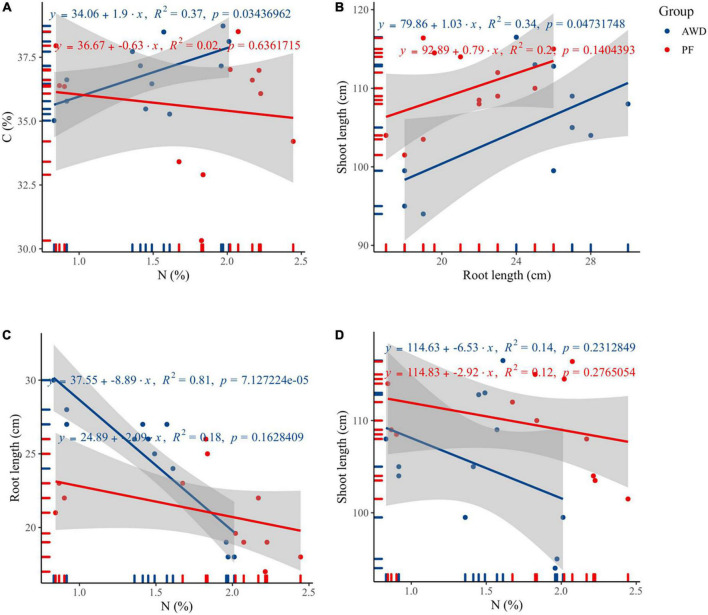
Correlation between **(A)** C and N, **(B)** shoot length and root length, **(C)** root length and nitrogen, and **(D)** shoot length and nitrogen during alternate wetting and drying (AWD) and permanent flooding (PF).

The N had a significant correlation with C (*R*^2^ = 0.37, *p* = 0.03; Figure 2A) and root length (*R*^2^ = 0.81, *p* < 0.000; Figure 2C), but no significant correlation of N was found with shoot length (*R*^2^ = 0.14, *p* = 0.23) during AWD (Figure 2D). Besides, the N correlations with C, root length, and shoot length were not significant during PF. Also, a significant correlation of shoot length was observed with root length during AWD (*R*^2^ = 0.34, *p* = 0.04), which showed AWD a determining factor for plant growth (Figure 2B).

### Grain yield

**FIGURE 3:**
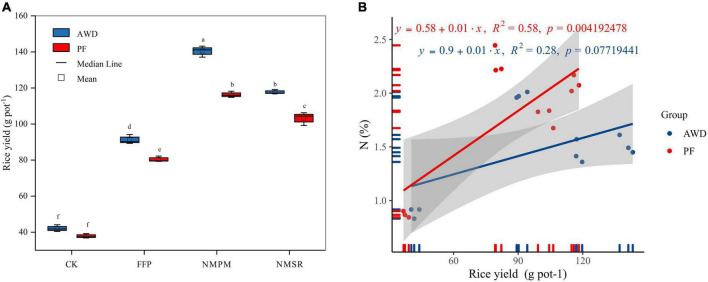
Combined effects of different fertilizer treatments on **(A)** rice yield (g pot^–1^) during alternate wetting and drying (AWD) and permanent flooding (PF). The arrow bar above the line shows standard error. The different letters show significant difference, while the same letters represent no significant differences among the treatments. **(B)** Correlation between rice yield and nitrogen concentration during AWD and PF water events.

Overall, grain yield ranged from 37.13 to 140.25 g pot^–1^; moreover, water events and fertilizer treatments significantly affected grain yield (Figure 3A). Compared to PF, total grain yield was increased by 15.97% in AWD. The lowest yield was observed in CK treatment under both AWD and PF events. When fertilizers were applied, FFP, NMPM, and NMSR significantly increased the yield during AWD and PF (*p* < 0.05) but relatively higher during AWD. The NMPM was found superior to FFP and NMSR in yield improvement regardless of water regime, yielding 9% more under AWD than PF (Figure 3A). Because of the increased effective panicles per pot, the combined PF and NMPM treatment obtained the best yield. Regardless of fertilization treatments, the yield was reduced by PF compared to AWD. However, significant differences were observed between both water events in FFP, NMPM, and NMSR, but there was no significant difference between both watering events in CK. Compared to FFP, NMPM increased rice yields by 54.6 and 45.6%, decreasing urea and pig manure by 42 and 71% under AWD and PF, respectively. Besides, a significant correlation was observed between grain yield and nitrogen content during AWD (*R*^2^ = 0.58, *p* < 0.01), but no significant correlation was observed during PF (Figure 3B) which showed N is very important in terms of grain yield under AWD.

### Photosynthetic parameters

**FIGURE 4:**
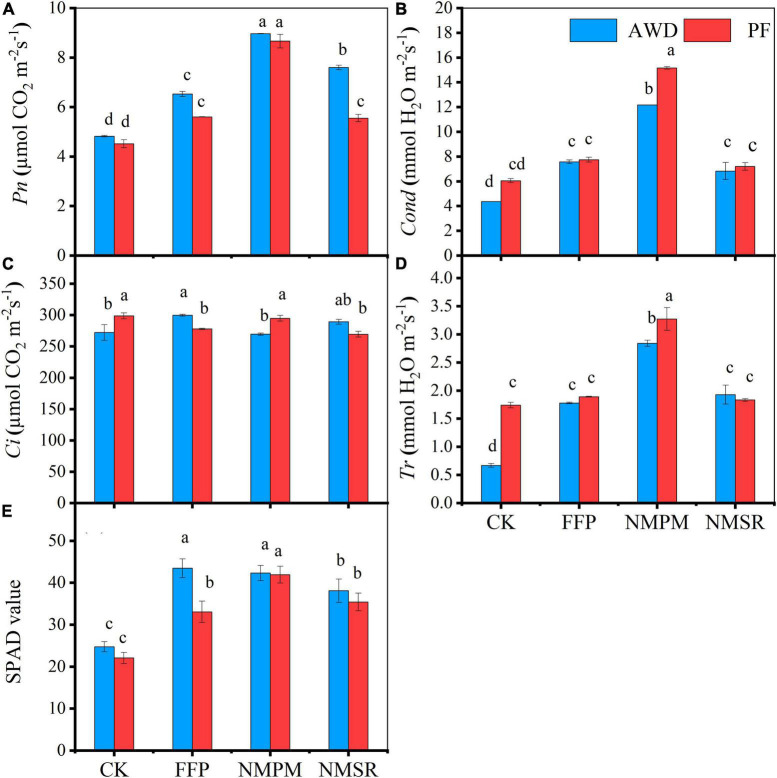
Combined effect of different fertilizer treatments and water level, alternate wetting and drying (AWD), and permanent flooding (PF) on photosynthetic parameters: **(A)** net photosynthesis rate, **(B)** stomatal conductance, **(C)** intercellular CO_2_, **(D)** transpiration rate, and **(E)** relative chlorophyll contents (photosynthetic SPAD values). The different letters show significant difference, while the same letters represent no significant differences among the treatments while arrow bars above the lines show standard error. *Pn; net photosynthesis rate, *Cond*; stomatal conductance, *Ci*; intercellular CO_2_, *Tr*; transpiration rate, SPAD; soil plant analysis development.

Rice plants treated with NMPM showed an increase in net *Pn* during AWD, but the difference between AWD and PF was not significant, while stomatal *Cond*, *Tr*, and intercellular CO_2_ *Ci* significantly increased during PF (Figure 4), suggesting that PF had a positive effect on *Cond*, *Tr*, and *Ci*. Meanwhile, the addition of FFP, NMPM, and NMSR to the soil significantly increased the *Pn* (Figure 4A). Overall, the net photosynthesis rate was lower in CK treatment inferring that fertilizer treatments promoted *Pn* (Figure 4A). The highest *Pn* rate was observed in NMPM followed by NMSR and FFP. While compared with CK, NMSR treatment showed the same *Pn* as FFP during PF, indicating that organic slow-release fertilizer took time to dissolve in the water. Other photosynthetic parameters (*Cond* and *Tr*) were also found higher in NMPM than in other fertilizer treatments, suggesting that low application rate of pig manure and N fertilizer still worked better than in higher doses (Figures 4B–D). AWD showed lesser values for other photosynthetic parameters too, implying that PF enhanced Cond, *Tr*, and *Ci* due to high water supply. No significant differences were observed in *Pn* between AWD and PF, but fertilizer treatments significantly differed from each other.

The SPAD value showed different trends in different fertilizer treatments during both water events (Figure 4E). During AWD, the highest SPAD value (42.33) was observed in NMPM treatment while the minimum (12.67) was observed in CK treatment, and the difference was four times higher. Furthermore, the highest value was observed during PF in NMPM followed by NMSR, FFP, and CK (Figure 4E). Inclusively, the highest SPAD values were measured during AWD than PF; meanwhile, NMPM contributed equally to both watering events. In comparison with control, significant increases were measured in fertilizer treatments during both conditions (*p* < 0.05), but the differences were not significant for water events.

### Nitrifier and denitrifier abundance

**FIGURE 5:**
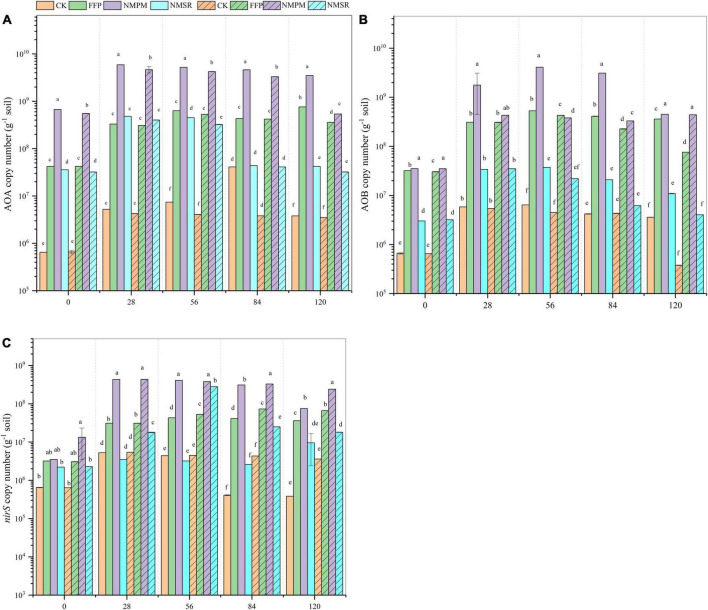
Abundance of **(A)** AOA *amoA*, **(B)** AOB *amoA*, and **(C)** *nirS* functional gene (copy numbers g dry soil^–1^) after 0, 28, 56, 84, and 120 days after transplantation during alternate wetting and drying (AWD) and permanent flooding (PF) events. Alternate wetting and drying is for alternating drying and flooding, while PF stands for permanent flooding. The panel without pattern shows AWD, while with pattern shows PF. The standard error (*n* = *3*) is shown by the arrow bar above the line.

The *amoA* AOA, *amoA* AOB, and *nirS* gene copy numbers were promoted with time under AWD and PF environment. A relatively higher abundance of the *amoA* AOA gene was recorded during AWD. The AOA abundance was observed higher after 28, 56, and 84 days in all treatments and then maintained or decreased. While comparing the fertilizer treatments, NMPM promoted *amoA* AOA, *amoA* AOB, and *nir*S gene abundances (Figure 5A). The *amoA* AOB abundance was found higher in NMPM treatment during AWD and PF but lower than AOA (Figure 5B). The abundance of the *amoA* AOB gene was observed in the range of 2.1E+05–4.3E+09 copy numbers g^–1^ of dry soil, with the peak value after 56 days under the NMPM treatment. A significant gradual increase in copy number of AOB was noted during AWD with time (*p* < 0.05). Also, during PF, a significant increase in copy number of AOB was observed with time in all treatments. During both water events, the abundance of the *nirS* gene grew dramatically with time, and significant differences were observed between treatments later in the experiment (*p* < 0.05). Furthermore, AOA, AOB, and *nirS* gene abundance were shown to be considerably greater in NMPM for the whole trial period in both water events. Despite the fact, the abundance of the *nirS* gene fluctuated over time under all moisture conditions (Figure 5C). Besides, the high gene abundance of *nirS* was found in the flooded condition in all fertilizer treatments, but a relatively higher abundance was in NMPM treatment.

## Discussion

Although AWD and NM techniques have been examined previously (Peng et al., 2010; Yao et al., 2012), the accompanying effects of AWD and NM on grain yield, and nitrifier and denitrifier abundance are still inconclusive. The high N input rate and improper timing of fertilizer application can cause lower NUE in irrigated Chinese rice (Peng et al., 2006; Ju et al., 2009). Most farmers apply N fertilizers at early vegetative growth (10 days after transplantation) which is not suitable for efficient use (Peng et al., 2010). On the contrary, water should also be applied according to the demand of the crop to maximize the water use efficiency. So, nitrogen fertilizer management in AWD irrigations is very important to increase their efficiencies.

### Effects of water events and fertilizers on inorganic nutrient concentration

Right application time and optimal N rate are considered beneficial to improve rice growth contributing characteristics. The application N rate in NM was reduced by 40% compared to FFP, especially at the vegetative growth stage. Our results showed a reduction of rice N concentration under NMPM and NMSR relative to FFP in vegetative growth (Table 1); however, total N concentration under NMSR exceeded FFP under PF because an increased N accumulation rate was observed for NMSR (Table 2), which might be due to fast and highest nutrient uptake at panicle initiation stage (Peng and Cassman, 1998). A higher concentration of N and other nutrients during PF might be due to a higher rate of diffusion. Similarly, N concentrations were significantly higher under FFP while lower in NMSR during AWD. Besides, high C concentration in root under CK treatment was due to the indigenous supply from the soil. While in shoot, C concentration was promoted by NMPM, which showed a high potential for C storage.

### Effects of water events and fertilizers on growth formation characters

Larger root dry matter and higher root activity involve in a high capacity for water and nutrient absorption, which favors high grain production (Zhang et al., 2009; Kato and Okami, 2010). The significantly higher root length was observed in AWD and had been reported previously (Kato and Okami, 2010), which is in line with current results displaying a positive root growth response under AWD (Figure 1), proposing water limitation is favorable for deeper root system (Kato et al., 2006). On the contrary, PF promoted the root biomass, consistent with Ndiiri et al. (2012). Besides, FFP treatment showed reduced root and shoot length and biomass, consistent with the previous findings (Kato and Okami, 2010). Efficient distribution of fertilizers resulted in higher plant growth in plants treated with NMPM and NMSR. Plant height was decreased in FFP treatments during both water events but increased in NMPM and NMSR (Figure 1C). However, the inhibition of plant growth differed greatly among the four fertilizer treatments, being highly affected by FFP and, to a lesser extent, by NMPM and NMSR contingent on the timely application of fertilizer (Figures 1A,C). The main reason for the increased shoot and whole plant biomass in flooded irrigation was adequate soil water before maturity (Figures 1C,D; Gong et al., 2005). Interestingly, root length had a significant correlation with N contents under AWD (Figure 2C) consistent with the previous studies (Zhang et al., 2009; Thakur et al., 2011), which showed that AWD caused a strong and healthy root system for nutrient uptake (Hazra and Chandra, 2016). Also, the number of tiller (Figure 1E) was enhanced by managed practices compared to the conventional practices (Zhang et al., 2020; Mboyerwa et al., 2021).

### Effects of water events and fertilizers on grain yield

According to the previous literature, different management practices have been employed successfully to enhance crop yield (Yang and Zhang, 2010; Liu et al., 2013). The results obtained from this study infer that both NMPM and NMSR, synergistically with AWD, increased grain yield by 20.59 and 14.07%, compared to the similar treatments under PF (Figure 3A). A significantly higher yield was observed under AWD (Figure 3A), suggesting that fertilizer treatments and AWD have a positive interaction and such an interaction could increase not only the grain yield but also the resource-use efficiency in rice (Liu et al., 2013). However, the underlying mechanism of nitrogen optimization in rice under AWD is not well-understood. We observed that either NMPM or AWD significantly increased the percent grain yield when NMPM and AWD have opted together (Figure 3A), owing to the reduction of water loss through transpiration from redundant leaf area, and nutrients loss by unproductive tiller growth. The incorporation of organic matter improved rice yield compared to slow-release fertilizer which stems from the lower efficiency of organic slow-release fertilizer due to delayed nutrient release. Besides, NMPM had a similar N rate as NMSR, but in terms of grain yield, NMPM performed better which could be attributed to better N distribution. These contradictory results might be due to the root elongation and absorption of N from soil under low N and water-saving conditions (Chu et al., 2014; Sun et al., 2014; Pan et al., 2017).

Reportedly, the increase in yield by AWD practice was an important finding of this study. Soil drying can enhance yield by increasing C remobilization and root enlargement for maximum nutrient uptake (Yang and Zhang, 2006; Li et al., 2016). The results showed a significant increase in root length under AWD than PF, while leaf photosynthetic rate and dry matter accumulation were markedly higher, particularly under both AWD and NM strategies (Figures 1, 4). A significant correlation was found between the root and shoot biomass (Figure 2B), suggesting that an improved root–shoot interaction under AWD-NM treatment positively contributed to higher grain yield. In contrast, an adequate water supply below the soil surface might also be a factor worth considering. Previous studies showed that −20 k Pa water potential was considered sufficient for the absorption of sufficient water from the soil (Lampayan et al., 2015; Carrijo et al., 2017). Hence, AWD is a better choice to maintain a high yield and conserve water. Similar grain yield results under AWD were reported previously (Cabangon et al., 2004; Yao et al., 2012).

The grain yield had a significant correlation with N content under AWD (Figure 3B), consistent with Zhang et al. (2019) and Zheng et al. (2020), which showed N can promote gain yield and agronomic traits of rice under AWD. Consistent results were found in ratoon crop in which N management promoted grain yield (Wang et al., 2021). The AWD improved oxygen supply to rice roots and causes a strong and healthy root system which improves nutrient uptake (Hazra and Chandra, 2016). Moreover, AWD promoted the activities of key enzymes involved in conversion of sucrose to starch in gain (Mboyerwa et al., 2022).

### Effects of water events and fertilizers on photosynthetic parameters

Nitrogen is an essential macro-nutrient, and its application rate and time are very important for plant productivity. The current results showed that the excessive N is highly detrimental to photosynthesis, but split and timely managed practices are more valuable, confirming the earlier findings (Yao et al., 2012), although the responses of photosynthesis rate to fertilizers also differed among water events, with AWD being less affected than PF (Figure 4A). In this study, NMSR significantly decreased SPAD value (relative chlorophyll content) (Figure 4E) and photosynthetic parameters; *Pn*, *Ci*, *Tr*, and CO_2_ exchange (Figures 4A,C,D) during PF but antagonistic results were found in CK and NMPM where PF promoted photosynthetic parameters and SPAD values. Comparatively, CK showed a very low photosynthesis rate during AWD compared to other fertilizer treatments which implied that all fertilizers promoted photosynthesis (Figure 4A). Higher N in terms of photosynthetic parameters may be assumed to be associated with a lower contribution in plant tissues. Alternatively, PF caused the increase of Cond and *Tr* values for all fertilizer treatments, although the SPAD value experienced nominal changes.

Photosynthetic rate responds reciprocally to increasing water contents (Jahan et al., 2014), gleaning water as an important substrate for this process. Still, the addition of fertilizer has augmentative effects by means of nutrient provision to the plants. Accordingly, our results showed the significant effects of fertilizer application on photosynthesis rate. Furthermore, photosynthesis decreases when the moisture contents in the soil reduce to field capacity. Because the moisture contents were above the field capacity level in this study, it did not impact the photosynthetic rate negatively. AWD reduced relative chlorophyll content in leaves through CO_2_ assimilation (Awal and Ikeda, 2002). These results found in control treatments were consistent with this study (Khairi et al., 2015) that plants accumulated lower relative chlorophyll content (Figure 4E) under AWD condition but did not find in fertilizer treatments (Figure 4E) and the results suggested the nutrients work better under combined AWD and PF conditions. Our results revealed that plants accumulate less relative chlorophyll content under AWD, which might be due to the effect of light-related growth and production (Jahan et al., 2014).

### Nitrifier and denitrifier abundance based on N fertilizer management

Functional genes of nitrifiers (*amoA* AOA and *amoA* AOB) and denitrifier (*nirS*) are crucial mediators of N turnover in paddy soils that affect rice growth by competing for the nutrients (Hussain et al., 2011). We observed the rice plant and rhizosphere genes interaction that affects N cycling. The gene copy numbers of *amoA* AOA, *amoA* AOB, and *nirS* were found higher in rhizosphere which was probably due to a rhizospheric effect since the rhizo-decomposition of carbohydrates favors microbial growth (Hussain et al., 2011). In this study, *amoA* AOA gene abundance was found more dominant than *amoA* AOB (Figures 5A,B), consistent with Chen et al. (2008). The *amoA* AOA, *amoA* AOB, and *nirS* gene abundances were found comparatively higher at grain-filling stage, attributing to enhanced root exudates and higher nutrients for microbial growth (Bürgmann et al., 2005). In accordance with our results, the *amoA* AOA always dominates in the paddy soil (Figure 5A) showing that AOA has better adaptation capacity than AOB to the microaerophilic environment in the rhizosphere and possesses higher affinity for ammonia or may get benefit from root exudates (Jung et al., 2011; Kits et al., 2017). Another reason for high nitrifier AOA abundance could be the release of rice roots through aerenchyma cells (Bedford et al., 1991).

Furthermore, the higher abundance of *amoA* AOA compared to AOB was attributed to AOA’s high affinity for oxygen (Jung et al., 2011; Straka et al., 2019). Previous research identified fertilization rate as the primary determinant of AOB and AOA abundances (Di et al., 2014). The availability of NH_4_^+^, which is generated by organic N mineralization or by the addition of inorganic NH_4_^+^, is thought to be the sole factor influencing the abundance of AOA and AOB (Tourna et al., 2008). However, the pH may alter NH_4_^+^ availability since it would be ionized to NH_4_^+^ at low pH (Nicol et al., 2008). As a result, fertilization appears to have a significant impact on increasing substrate availability for AOA in a paddy field.

The abundance of *nirS* was lower than that of nitrifiers, which is consistent with Yoshida et al. (2009). During AWD, the abundances of the *nirS* gene grew rapidly throughout time, but in PF, the abundances increased until 84 days and then stabilized or began to fall (Figure 5C). The decrease in *nirS* during the latter phases might be attributed to nutritional insufficiency or absolute anaerobic conditions (Uchida et al., 2014). Furthermore, since denitrifier development is often encouraged under anaerobic circumstances, the flooding-drying cycle affected denitrifier abundance (Di et al., 2014; Uchida et al., 2014). Alternatively, Cui et al. (2016) observed a significant rise in denitrifier abundance (*nirS*) following long-term fertilization, which is comparable to our findings, where denitrifier abundance was shown to be high under NMPM and FFP treatments (Figure 5C). The increased copy number of *nirS* might be attributed to dissolved organic carbon (DOC), NO_3_^–^ and NO_2_^–^, all of which are responsible for the rise in denitrifier copy numbers in fertilized soils (Hamonts et al., 2013).

## Conclusion

Alternate wetting and drying has been widely adopted to replace submerged irrigation in irrigated rice systems to increase grain yield, NUE, and photosynthesis. It could be deduced from the current results that AWD is a better choice to save water without sacrificing grain yield as it resulted in similar or better biomass, grain yield, and NUE than permanent flooding. While comparing fertilizer treatments, NMPM was observed superior in terms of rice yield and NUE. In addition, NMPM works nearly equally during both watering events. The use of slow-release fertilizer in this study, which is a better choice than conventional FFP, fell short compared to the efficiency of NMPM. Compared to FFP and conventional submerged irrigation PF, NMPM (chemical N replaced by organic N based on the NMPM-AWD model) works comparatively better. Further investigations are required to analyze how the NMPM-AWD model works in different environments and soils. Also, studies regarding the impact of NMPM-AWD model on soil microbial ecology are much needed.

## Data availability statement

The original contributions presented in this study are included in the article/Supplementary material, further inquiries can be directed to the corresponding author.

## Author contributions

AA: writing—original draft preparation, conceptualization, and formal analysis. QZ: conceptualization, resources, supervision, and writing—reviewing and editing. IB, MA, and MFA: methodology, software, and writing. AC-H: reviewing and editing and software. HD: reviewing and editing. All authors contributed to the article and approved the submitted version.
