# Supplementary material for: Nitrogen optimization coupled with alternate wetting and drying practice enhances rhizospheric nitrifier and denitrifier abundance and rice productivity
Source: Front Plant Sci. 2022 Oct 11;13:927229. doi: 10.3389/fpls.2022.927229 (PMC9592971; doi:10.3389/fpls.2022.927229)
Supplement: Supplementary file 1 [file Table_1.DOCX]

**Supplementary materials**

**Nitrogen optimization coupled with alternate wetting and drying practice enhance rhizospheric nitrifier and denitrifier abundance, and rice productivity**

Abbas Ali Abid^1^, Qichun Zhang^1^*, Muhammad Faheem Adil^2^, Itrat Batool^3^, Muhammad Abbas^4^, Zeshan Hassan^5^, Azhar Abbas Khan^5^, Antonio Castellano-Hinojosa^6^, Syed Hassan Raza Zaidi^2^, Hongjie Di^1^, Nader R. Abdeslsalam^7^

^1^Zhejiang Provincial Key Laboratory of Agricultural Resources and Environment, Key Laboratory of Environment Remediation and Ecological Health, Ministry of Education, Zhejiang University, Hangzhou, 310058, P.R China

^2^Department of Agronomy, Institute of Crop Science, College of Agriculture and Biotechnology, Zhejiang University, Hangzhou 310058, P.R China

^3^Institute of Food Science, Chinese Academy of Agricultural Sciences, Beijing, China

^4^National Engineering Laboratory for Improving Quality of Arable Land. Institute of Agricultural Resource and Planning, Chinese Academy of Agricultural Sciences, Beijing, China

^5^College of Agriculture, Bahauddin Zakariya University, Bahadur Campus, Layyah 31200, Pakistan

^6^Department of Soil and Water Sciences, Southwest Florida Research and Education Center, Institute of Food and Agricultural Sciences, University of Florida, Immokalee, FL 34142, USA

^7^Agriculture Botany Department, Faculty of Agriculture, Saba Basha, Alexandria University, Alexandria, 21531, Egypt

***Corresponding author:**

Dr. Qichun Zhang

College of Environmental and Resource Sciences,

Zhejiang University,

Hangzhou 310058,

P.R China

Phone: +86-571-88982413

Email: [qczhang@zju.edu.cn](mailto:qczhang@zju.edu.cn)

| **Table S1:** Real-time PCR primer sets, conditions of the assay | | | | | |
| --- | --- | --- | --- | --- | --- |
| Target gene Base pairs | | Primer | Nucleotide sequence (5'-3') | Annealing temperature and time | Reference |
| Bacterial *amo*A | 491 bp | *amoA*-1F | GGGGTTTCTACTGGTGGT | 55 °C for 45 s | Rotthauwe et al.1997 |
|  |  | *amoA*-2R | CCCCTCKGSAAAGCCTTCTTC |  |  |
| Archaeal *amo*A | 635 bp | *amoA*F | STAATGGTCTGGCTTAGACG | 53 °C for 45 s | Francis et al. 2005 |
|  |  | *amoA*R | GCGGCCATCCATCTGTATGT |  |  |
| *nir*S | 425 bp | Cd3aF | GTSAACGTSAAGGARACSGG | 57 °C for 30 s | [Throbäck et al. 2004](http://www.sciencedirect.com/science/article/pii/S0929139317303438#bib0225) |
|  |  | R3Cd | GASTTCGGRTGSGTCTCTTGA |  |  |

**References**

1. Francis, C.A., Roberts, K.J., Beman, J.M., Santoro, A.E., Oakley, B.B. 2005. Ubiquity and diversity of ammonia-oxidizing archaea in water columns and sediments of the ocean. Proc. Natl. Acad. Sci. 102, 14683–14688.
2. Rotthauwe, J.H., Witzel, K.P., Liesack, W. 1997. The ammonia monooxygenase structural gene *amoA* as a functional marker: molecular fine-scale analysis of natural ammonia-oxidizing populations. Appl. Environ. Microbiol. 63, 4704–4712.
3. Throbäck, I.N., Enwall, K., Jarvis, A., Hallin, S. 2004. Reassessing PCR primers targeting *nirS*, *nirK* and *nosZ* genes for community surveys of denitrifying bacteria with DGGE. FEMS Microbiol. Ecol. 49, 401–417.
